# Increased Macrolide Resistance Rate of M3562 *Mycoplasma pneumoniae* Correlated With Macrolide Usage and Genotype Shifting

**DOI:** 10.3389/fcimb.2021.675466

**Published:** 2021-05-12

**Authors:** Yacui Wang, Baoping Xu, Xirong Wu, Qingqin Yin, Yi Wang, Jieqiong Li, Weiwei Jiao, Shuting Quan, Lin Sun, Yonghong Wang, Adong Shen

**Affiliations:** ^1^ Beijing Key Laboratory of Pediatric Respiratory Infection Diseases, Key Laboratory of Major Diseases in Children, Ministry of Education, National Clinical Research Center for Respiratory Diseases, National Key Discipline of Pediatrics (Capital Medical University), Beijing Pediatric Research Institute, Beijing Children’s Hospital, Capital Medical University, National Center for Children’s Health, Beijing, China; ^2^ Department of Respiratory Medicine, Beijing Children’s Hospital, Capital Medical University, National Center for Children’s Health, Beijing, China; ^3^ Experimental Research Center, Capital Institute of Pediatrics, Beijing, China; ^4^ Children’s Hospital Affiliated to Zhengzhou University Henan Children’s Hospital Zhengzhou Children’s Hospital, Zhengzhou, China

**Keywords:** *Mycoplasma pneumoniae*, macrolide resistance, genotype, disease severity, pediatrics

## Abstract

To characterize *Mycoplasma pneumoniae* (MP) strains and to clarify the continuous high rates of macrolide resistance, 1,524 oropharyngeal swabs collected from children in Beijing Children’s Hospital infected with MP during 2016-2019 were analyzed. Among the 1,524 samples, 1,386 harbored mutations associated with macrolide resistance; 1,049 samples were successfully classified into 11 genotypes using multiple locus variable-number tandem-repeat analysis (MLVA). The proportion of the predominant type, M4572, decreased from 84.49 to 70.77% over the time period examined, while that of M3562 increased from 11.63 to 24.67%. Notably, we also found that the frequency of macrolide resistance in M3562 drastically increased, from 60% in 2016 to 93.48% in 2019. Clinical data suggested that the frequency of resistant M3562 was higher in the macrolide usage group than in the nondrug usage group (90.73 vs 53.57%, P<0.0001), while the resistance rate of M4572 was not substantially affected by previous macrolide exposure. These findings validated that antimicrobial application and clonal expansion of resistant MP strains play important roles in the high rates of macrolide resistance.

## Introduction

*Mycoplasma pneumoniae* (MP) is an established causative agent of respiratory infection, accounting for 20-40% of community-acquired pneumonia cases in pediatric patients (Qian and Wei, 2016; Gao et al., 2019). The clinical manifestations of MP infection are highly variable, ranging from mild respiratory infection symptoms to severe pneumonia, as well as extrapulmonary complications that can be fatal in some circumstances.

Macrolides are recommended as the first-choice antibiotics for Mycoplasma pneumoniae pneumonia (MPP) treatment in children, as alternative antibiotics such as tetracycline and quinolone are not commonly recommended for use in children (Bradley et al., 2011; The Committee of Japanese Society of Mycoplasmology, 2014; Respiratory Branch of Chinese Pediatric Society of Editorial Board of Chinese Journal of Applied Clinical Pediatrics, 2015; Pneumonia (community-acquired): antimicrobial prescribing, 2019). However, macrolide-resistant MP (MRMP) strains have emerged since 2000 and are increasing rapidly worldwide, with high rates of resistance of approximately 80-90% witnessed in Asia, especially in China and Japan (Zhao et al., 2012; Kawai et al., 2013). Notably, Japan witnessed a remarkable reduction in macrolide resistance after 2011-2012 when an outbreak of MP infection was observed (Okada et al., 2012), and the MRMP rates even dropped to 11.3% during 2018-2019 (Morozumi et al., 2020). In contrast, the proportion of MRMP was maintained at a high level during 2013-2018 in China (Xue et al., 2018; Yan et al., 2019; Zhao et al., 2019). Owing to the limited drugs available for MPP treatment in children, the emergence of MRMP strains makes the treatment more complicated in pediatric patients. Given that MRMP strains dominate in China and complicate the treatment in children, further investigations are warranted to continuously monitor the incidence of MRMP strains and to identify factors contributing to high rates of macrolide resistance.

Multiple locus variable-number tandem-repeat analysis (MLVA), a genotyping method with a more powerful discriminatory ability than previous typing methods based on the P1 gene, provides the best way to characterize strains isolated during infection and to investigate the correlation between genotypes and drug resistance. Previous studies have established an association between macrolide resistance and specific MLVA genotypes (Zhou et al., 2014; Zhao et al., 2019), while related research is relatively limited, and further explorations are needed. In addition, macrolide usage has also been reported to be correlated with drug resistance (Morozumi et al., 2008; Lee et al., 2018).

In this report, MLVA typing was conducted to characterize MP strains in clinical specimens collected from children who were enrolled in Beijing Children’s Hospital from 2016 to 2019 and diagnosed with MPP, and to analyze the relationship between macrolide resistance and specific genotypes. Then, to better understand consistently high rates of drug resistance, clinical information on macrolide usage prior to hospital admission was extracted from medical records and analyzed.

## Materials and Methods

### Patients and Clinical Specimens

A total of 1,524 oropharyngeal swabs, obtained from hospitalized children diagnosed with MPP in Beijing Children’s Hospital between January 2016 and September 2019, were analyzed. Most of the patients were living in northern China. MPP was diagnosed using the following criteria: symptoms of acute fever, cough, and other respiratory manifestations; rales on auscultation; changes of consolidation, infiltration and interstitial on chest radiograph; and positive PCR results for MP. Severe MPP (SMPP) was defined as MPP with one of the following characteristics (Subspecialty Group of Respiratory Diseases. The Society of Pediatrics, 2013): (1) poor general condition; (2) increased breathing rate; (3) cyanosis and dyspnea; (4) infiltration involving multiple lobes or ≥2/3 of the lung; (5) transcutaneous oxygen saturation ≤92% in room air; and (6) extrapulmonary complications. Repeated specimens collected from the same patient were excluded from the study. All samples collected in this study were part of the routine engagement of patients without additional collection. Information on demographic characteristics, prehospital macrolide usage, and disease severity was gathered from the clinical records.

This study was approved by the Beijing Children’s Hospital Ethics Committee. Informed consent for participation in the study was obtained from the patients or the guardians.

### Identification of MP and Macrolide Resistance-Associated Mutations in Domain V of 23S rRNA

Real-time PCR testing was initiated by clinicians to identify the presence of MP and MRMP using a Mycoplasma pneumoniae and macrolide-resistant isolates diagnostic kit (Mole, Jiangsu, China) as described previously (Feng et al., 2016). In brief, the P1 gene was detected by a real-time PCR assay to confirm MP infection, and domain V of 23S rRNA in MP was detected to identify mutations responsible for macrolide resistance. MP-positive DNA remnants were stored at -80°C for further MLVA typing analysis.

### MLVA Typing

MLVA genotyping was conducted based on a previously described culture-independent method with slight modifications (Dumke and Jacobs, 2011). Briefly, nested PCR was conducted using previously published primers, targeting the four selected variable-number tandem-repeat (VNTR) loci (Mpn13, Mpn14, Mpn15, Mpn16) (Dumke and Jacobs, 2011; Yan et al., 2014). Then, the PCR products were sequenced and the VNTR copy numbers were calculated to define the MLVA types (Degrange et al., 2009).

### Statistical Analysis

SPSS Statistics for Windows (version 23.0) was used for statistical analysis. Chi-square or Fisher’s exact test was used for categorical analysis, and Student’s t test was used for continuous data. A p value of < 0.05 was considered significant.

## Results

### General Information of the Patients

A total of 1,524 patients diagnosed with MPP by real-time PCR were enrolled in the study. The proportions of patients with MPP by year were as follows: 16.67% in 2016, 23.16% in 2017, 30.58% in 2018, and 29.59% in 2019. The average age of the patients was 6.83 years; 15.35% (234/1,524) of the patients were, aged ≤3 years, 17.26% (263/1,524) were aged 3-5 years, 62.99% (960/1,524) were aged 5-12 years, and 4.40% (67/1,524) were aged 12-18 years. MPP was more prevalent among children 5-12 years of age than among other age groups. Overall, 54.07% (824/1,524) of the participants were male, and 45.93% (700/1,524) were female, with a female to male ratio of 1.18 (**Table 1**).

**Table 1:** Demographic data of MP-positive patients.

General information	MP-positive patients (n=1524)	Ratio (%)
Age, y		
0-3	234	15.35
3-5	263	17.26
5-12	960	62.99
12-18	67	4.40
Sex		
M	824	54.07
F	700	45.93
Macrolide usage prior to hospital*		
Yes	1,336	88.36
No	176	11.54
SMPP		
Yes	514	33.73
No	1,010	66.27

*Twelve cases with incomplete information of macrolide usage prior to hospital were excluded.

### Detection of Macrolide Resistance-Associated Mutations in the 23S rRNA Gene

Macrolide resistance detection was successfully conducted for all 1,524 MP-positive specimens. Overall, 90.94% (1,386/1,524) of the MP samples were macrolide-resistant (MRMP), and 9.06% (138/1524) were macrolide-sensitive without mutation (MSMP). The annual prevalence of MRMP was as follows: 88.19% (224/254) in 2016, 90.93% (321/353) in 2017, 90.56% (422/456) in 2018, and 92.9% (419/451) in 2019.

### MLVA Typing

**Figure 1:**
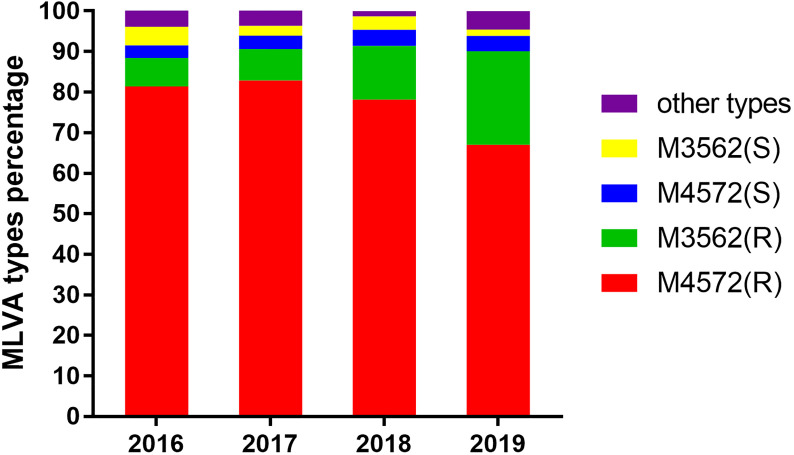
The distribution of different MLVA types of MP from 2016 to 2019 (N=1,049).

Of 1,524 clinical samples with MP infection, 475 specimens were unable to be profiled by MLVA, because some essential amplicons corresponding to target genes were not obtained for MLVA genotyping. Thus, 1,049 clinical samples were successfully analyzed by the MLVA method, and 11 different types, including M4572, M3562, M3572, M3662, M4472, M4552, M4562, M4571, M4573, M4662, and M4672, were generated. The distribution characteristics of the MLVA types are shown in **Figure 1**. Two MLVA types, including M4572 (79.31%, 832/1,049) and M3562 (17.35%, 182/1,049), were predominant and constantly present during the period of this study. Other rare genotypes identified by MLVA only accounted for 3.35% of the total (**Table 2**). Stratified by year, M3562 type significantly increased from 11.63% in 2016 to 24.67% in 2019 (P<0.0001), whereas the M4572 type declined from 84.49% to 70.77% in the same time frame (P<0.0001) (**Figure 1**).

**Table 2:** MLVA types and macrolide resistance.

MLVA types	No. specimens (%)	Macrolide resistance (%)	
		Resistant (n=980)	Sensitive (n=69)
M3562	182/1,049 (17.35)	154/182 (84.62)	28/182 (15.38)
M3572	6/1,049 (0.57)	5/6 (83.33)	1/6 (16.67)
M3662	4/1,049 (0.38)	4/4 (100.00)	0 (0.00)
M4472	3/1,049 (0.29)	3/3 (100.00)	0 (0.00)
M4552	4/1,049 (0.38)	4/4 (100.00)	0 (0.00)
M4562	3/1,049 (0.29)	3/3 (100.00)	0 (0.00)
M4571	1/1,049 (0.10)	1/1 (100.00)	0 (0.00)
M4572	832/1,049 (79.31)	794/832 (95.43)	38/832 (4.57)
M4573	3/1,049 (0.29)	2/3 (66.67)	1/3 (33.33)
M4662	1/1,049 (0.10)	0 (0.00)	1/1 (100.00)
M4672	10/1,049 (0.95)	10/10 (100.00)	0 (0.00)

### Relationship Between Macrolide Resistance-Associated Mutations and MLVA Types

**Figure 2:**
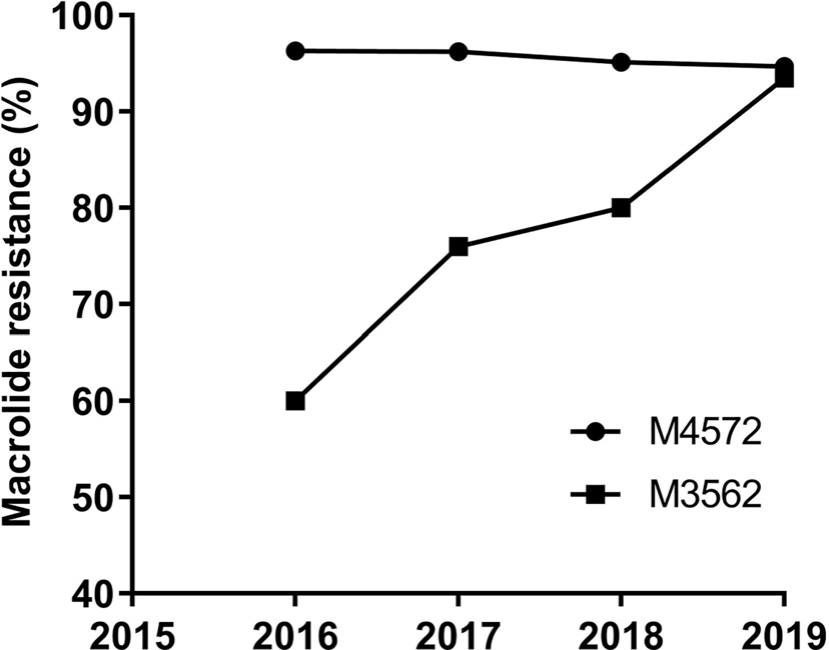
Macrolide resistance rates of MP with M4572 and M3562 types between 2016 and 2019 (N=1,014).

Of the eleven identified MLVA types, ten and five were found in the MRMP and MSMP groups, respectively (**Table 2**). The two main MLVA types, M4572 and M3562, occurred at different frequencies among the MRMP and MSMP groups. The prevalence of the M4572 type was significantly higher in the MRMP group than in the MSMP group (81.02% vs 55.07%, respectively; P<0.0001). In contrast, genotype M3562 was more prevalent in the MSMP group than in the MRMP group (40.58% vs 15.71%, respectively; P<0.0001). Stratified by year, macrolide resistance rates of the M4572 type remained relatively high, exceeding 90%, while MRMP with type M3562 increased significantly from 60.00% in 2016 to 93.48% in 2019 (P=0.02) (**Figure 2**).

### The Relationship Between Macrolide Usage and Macrolide Resistance-Associated Mutations

We examined the correlation between macrolide usage before enrollment and drug resistance of MP in clinical specimens. Twelve subjects with unknown antibiotic history were excluded. Among the remaining 1,512 MP-infected patients, 1,336 had taken macrolides prior to visiting the hospital, and 99.2% possessed mutations in the 23S rRNA gene compared to 81.25% of those in 176 infected patients who had not taken macrolides (P<0.0001). Among 179 subjects infected with the M3562 type, MRMP was more prevalent in the macrolide usage group than in the non-macrolide usage group (90.73 vs 53.57%, P<0.0001) (**Table 3**). Among 828 subjects infected with the M4572 type, 748 had taken macrolides before admission and 95.86% of those samples had mutations compared with 91.25% of 80 infected patients who had not taken macrolides, and no significant difference was identified (P=0.061) (**Table 3**). The average duration of treatment among the macrolide usage group prior to hospitalization was 5.59 days for all 1,336 patients, 5.81 days for patients infected with type M3562, and 5.82 days for patients with type M4572 (data not shown).

**Table 3:** The influence of prehospital macrolide usage on drug resistance and the distribution of severe MPP within M4572 and M3562 types *.

Genotypes	MRMP % (No)		P	Severe MPP %(No)	P
	Macrolide usage	Non-macrolide usage			
M3562	90.73 (137/151)	53.57 (15/28)	<0.0001	34.07 (62/182)	0.989
M4572	95.86 (717/748)	91.25 (73/80)	0.061	34.01 (283/832)	

*A total of 1,014 patients infected with MP classified as type M4572 and M3562, of which 7 cases with unknown medication information were excluded.

### The Relationship Between MLVA Type and Disease Severity

Of the 1,524 cases investigated, 33.73% (514/1,524) were diagnosed with SMPP. The proportion of SMPP each year was as follows: 37.01% (94/254) in 2016, 43.63% (154/353) in 2017, 28.33% (132/466) in 2018, and 29.71% (134/451) in 2019. The clinical relevance of MLVA types was analyzed with types M4572 and M3562 because the remaining types were not sufficient for the analysis. As shown in **Table 3**, there was no statistically significant difference in the incidence of SMPP between patients infected with type M4572 and those infected with type M3562 (34.01 vs 34.07%, P=0.989).

## Discussion

Our study reported high rates of macrolide resistance in recent years, similar to findings from previously published data in China (Sun et al., 2017; Yan et al., 2019). It is well known that macrolide resistance rates have remained high in Asia, such as in South Korea (Lee et al., 2018). However, the incidence of macrolide-resistant MP strains has drastically decreased in Japan in recent years (Morozumi et al., 2020). The rate of macrolide resistance was quite low in European countries (1-19%) (Uldum et al., 2010; Cardinale et al., 2013; Ferguson et al., 2013; Pereyre et al., 2013; Kogoj et al., 2015) and in the United States (8.3-10%) (Diaz et al., 2015; Xiao et al., 2020). The frequency of macrolide usage plays an important role in the difference in resistance rates among different countries. As Hsia reported, the most commonly used antibiotic for hospitalized children in China, Japan, and South Korea is azithromycin, with a prescription rate of 11.8%; while the prescription rate of this drug in Europe, America, Africa and Southeast Asia is only 1.9% (Hsia et al., 2019).

The distribution of MLVA types differed over time and among different geographic regions. The present study demonstrated that there was a type shift phenomenon from type M4572 to type M3562 in northern China, similar to the findings from previous reports between 2003 and 2018 in Beijing, which also reported a type change trend from M4572 to M3562 (Ho et al., 2015; Sun et al., 2017; Yan et al., 2019; Zhao et al., 2019). As previously reported in Japan, an alternative type-shift phenomenon of MLVA types occurred at an interval of approximately 10 years (Kenri et al., 2008). Therefore, we speculated that the type shift pattern of MP in northern China follows that of Japan, which means that China will see the predominance of M3562 in the next few years. However, the tendency of MLVA type changes in other parts of the world, except Japan (Suzuki et al., 2017), and the United States (Diaz et al., 2015; Xiao et al., 2020), contrasts somewhat with China. There was a type shift occurring worldwide approximately between 2011-2014: a shift from M4572 to M3562 in China (Zhao et al., 2013; Sun et al., 2017; Yan et al., 2019; Zhao et al., 2019), Japan (Suzuki et al., 2017), and the United States (Diaz et al., 2015; Xiao et al., 2020); and a shift from M3562 to M4572 in Slovenia (Kogoj et al., 2018), Sweden (Gullsby et al., 2019), and Russia (Voronina et al., 2020). The above changes in the dominance of MLVA subtypes over time may reflect the natural shifts of types, and the difference in the trend of type changes among distinct regions probably depends on the genotype initially introduced in the region.

Of note, a substantial increase in the prevalence of MRMP genotyped as M3562 was observed in this research. To date, MRMP has been acknowledged to be associated with specific MLVA types. Scientists in previous studies have suggested that genotype M4572 is closely related to macrolide resistance, while M3562 is related to macrolide susceptibility (Qu et al., 2013; Zhao et al., 2013). In this study, our findings indicated that the overall rate of macrolide resistance in the M3562 type was dramatically increased, compared with preliminary results (Sun et al., 2017; Zhao et al., 2019). Macrolide usage and type shifting may account for this phenomenon. Antimicrobial use served as a major driving force for the development of MRMP within the M3562 type, as it can be concluded from our data that patients infected with MRMP strains of the M3562 type more frequently received macrolides before hospitalization. These results indicated that the development of MRMP with the M3562 type was closely associated with macrolide application. Genotype shifting from type M4572 to M3562 was the main factor contributing to the continuous increase in MRMP rates within the M3562 type. It is known that in China, MP strains classified as the M3562 type were relatively low until 2013 (Zhao et al., 2013; Xue et al., 2014; Sun et al., 2017), and were thus rarely exposed to macrolides, resulting in a low incidence of MRMP with type M3562. However, with the type switching from M4572 to M3562, more MRMP strains with type M3562 have been successfully selected for and survived under tremendous antimicrobial pressure, and the selected MRMP strain developed an ability to spread rapidly in the population, leading to a large increase in the prevalence of MRMP with the M3562 type. Likewise, Japan has witnessed an upsurge in MP strains of the M3562 type since 2011, while MRMP rates of this type were relatively low compared with those reported in China (Suzuki et al., 2017). Clearly, macrolide prescription became more strict in Japan after the publication of the guidelines for MPP treatment in 2011, which may have contributed to the current low rate of MRMP (Morozumi et al., 2020). Taken together, we can conclude that antimicrobial application was a key factor in the development of MRMP. More evidence has confirmed that patients infected with MRMP suffer from longer durations of therapy and poor clinical response (Cao et al., 2010; Yang et al., 2019); therefore, macrolides should be used with caution in clinical settings.

Regarding type M4572, our research demonstrated that the frequency of this type was decreased over the time period, while MRMP rates remained at a high level. Based on the data of macrolide usage prior to hospitalization, the resistance rate with M4572 did not differ between the macrolide usage group and the non-macrolide usage group, in contrast to what was observed in type M3562, for which macrolide application was critical for the marked increase in MRMP rates, indicating a weak correlation between macrolide usage and the current high rates of MRMP within M4572. Isolates classified as type M4572 have been dominantly circulating in China for nearly 20 years (Zhao et al., 2013; Sun et al., 2017; Yan et al., 2019); MRMP strains of this type were successfully selected and survived after a long-term interaction with macrolides. Consequently, almost all the strains with type M4572 currently circulating in China were resistant to macrolides. Taken together, we deduced that the present high rates of MRMP with type M4572 predominantly resulted from a rapid clonal dissemination of MRMP strains of this type. Likewise, clonal expansion of macrolide-resistant strains of a specific type has also been reported in Hong Kong (Zhao et al., 2013), Taiwan (Hung et al., 2020), Japan (Suzuki et al., 2017), and South Korea (Lee et al., 2018). Given that type M3562 is currently increasing in several countries, measures should be taken to prevent the global expansion of this clone.

Notably, the occurrence of SMPP has been reported to have increased in China in recent years (Gao et al., 2019), and poses a great threat to children’s health. Our data suggested that SMPP made up 33.73% of all MP-positive cases, which was higher than that reported in previous publications (Gao et al., 2019). However, there are limited data investigating the clinical relevance of MLVA types. Our findings revealed that there were no type-based differences in the prevalence of SMPP between M4572 and M3562, suggesting the virulence of the strains with type M4572 and M3562 may be unremarkable. These observations coincided with what was reported in Spain where no correlation between MLVA types and clinical symptoms was noted (Rivaya et al., 2020). However, contrary to the above reports, some clinical studies have found that certain MLVA types were associated with disease severity, and that the pathogenicity of MP varied among different MLVA types (Qu et al., 2013; Yan et al., 2019). As reported in earlier studies, Qu et al. found that patients infected with the M4572 type had more severe disease than those infected with other types (Qu et al., 2013), yet scientists in another study have suggested that isolates clustered at the M3562 type exhibit a higher risk of progressing to SMPP (Yan et al., 2019). Currently, the clinical relevance of MLVA types has not been elucidated; therefore, further investigations are warranted to establish the correlation between MLVA type and clinical characteristics.

Our research had several limitations. First, the regions where patients were enrolled were limited, and therefore, the findings obtained in this study might not represent the reality of the whole country. Second, all subjects were inpatients, which may have contributed to the higher rates of SMPP, and the clinical and molecular characteristics of outpatients with MP infection are warranted for further investigations. Third, the number of patients who had not used macrolides prior to hospitalization was small, and more cases without macrolide application before admission are needed to further characterize the correlation between drug usage and the rate of macrolide resistance.

## Conclusion

In summary, this study demonstrated that the prevalence of macrolide-resistant MP strains was relatively high in pediatric patients in northern China. Additionally, a remarkable increase in MRMP strains classified as type M3562 throughout the study period was noted. Moreover, our findings suggested that there was a type shift from M4572 to M3562. Comprehensive data of genotyping and clinical information data demonstrated that high rates of macrolide resistance of type M3562 were associated with macrolide usage and genotype shifting, and that clonal expansion of MRMP strains was related to higher M4572 drug resistance.

## Data Availability Statement

The datasets presented in this study can be found in online repositories. The data is deposited in the GenBank repository, and the accession numbers are: MW917245 to MW921440.

## Ethics Statement

The studies involving human participants were reviewed and approved by Beijing Children’s Hospital Ethics Committee. Written informed consent to participate in this study was provided by the participants’ legal guardian/next of kin.

## Author Contributions

AS, YCW, YW, JL, and WJ conceptualized and designed the study. YCW, YW, and AS wrote the manuscript. BX, XW, and QY enrolled the subjects and collected data and samples. SQ, YHW, LS, and YCW performed the tests and analyzed the data. All authors approved the final manuscript as submitted and agree to be accountable for all aspects of the work. All authors contributed to the article and approved the submitted version.

## Funding

This study was supported by the Science and Technology Project of Beijing for the glucocorticoid therapy study of pediatric severe mycoplasma pneumoniae pneumonia (Z171100001017081), the Beijing Scientific Research Foundation for Returned Overseas Chinese Scholars, and the Beijing Young Talents Project (2016000021223ZK38).

## Conflict of Interest

The authors declare that the research was conducted in the absence of any commercial or financial relationships that could be construed as a potential conflict of interest.

## References

[B1] Bradley J. S. Byington C. L. Shah S. S. Alverson B. Carter E. R. Harrison C.. (2011). The Management of Community-Acquired Pneumonia in Infants and Children Older Than 3 Months of Age: Clinical Practice Guidelines by the Pediatric Infectious Diseases Society and the Infectious Diseases Society of America. Clin. Infect. Dis. 53, e25–e76. 10.1093/cid/cir531 21880587 PMC7107838

[B2] Cao B. Zhao C. J. Yin Y. D. Zhao F. Song S. F. Bai L.. (2010). High Prevalence of Macrolide Resistance in *Mycoplasma Pneumoniae* Isolates From Adult and Adolescent Patients With Respiratory Tract Infection in China. Clin. Infect. Dis. 51, 189–194. 10.1086/653535 20540621

[B3] Cardinale F. Chironna M. Chinellato I. Principi N. Esposito S. (2013). Clinical Relevance of *Mycoplasma Pneumoniae* Macrolide Resistance in Children. J. Clin. Microbiol. 51, 723–724. 10.1128/JCM.02840-12 23224091 PMC3553869

[B4] Degrange S. Cazanave C. Charron A. Renaudin H. Bebear C. Bebear C. M. (2009). Development of Multiple-Locus Variable-Number Tandem-Repeat Analysis for Molecular Typing of *Mycoplasma Pneumoniae* . J. Clin. Microbiol. 47, 914–923. 10.1128/JCM.01935-08 19204097 PMC2668363

[B5] Diaz M. H. Benitez A. J. Winchell J. M. (2015). Investigations of *Mycoplasma Pneumoniae* Infections in the United States: Trends in Molecular Typing and Macrolide Resistance From 2006 to 2013. J. Clin. Microbiol. 53, 124–130. 10.1128/JCM.02597-14 25355769 PMC4290910

[B6] Dumke R. Jacobs E. (2011). Culture-Independent Multi-Locus Variable-Number Tandem-Repeat Analysis (MLVA) of *Mycoplasma Pneumoniae* . J. Microbiol. Methods 86, 393–396. 10.1016/j.mimet.2011.06.008 21704086

[B7] Feng X. L. Li Q. Q. Sun L. Jiao W. W. Xu B. P. Yin J.. (2016). The Clinical Characteristics of Macrolide-Resistant *Mycoplasma Pneumoniae* Pneumonia in Children: A Case-Control Study. Chin. J. Evid. Based Pediatr. 11, 357–360. 10.3969/j.issn.1673-5501.2016.05.008

[B8] Ferguson G. D. Gadsby N. J. Henderson S. S. Hardie A. Kalima P. Morris A. C.. (2013). Clinical Outcomes and Macrolide Resistance in *Mycoplasma Pneumoniae* Infection in Scotland, UK. J. Med. Microbiol. 62, 1876–1882. 10.1099/jmm.0.066191-0 24008501

[B9] Gao L. W. Yin J. Hu Y. H. Liu X. Y. Feng X. L. He J. X.. (2019). The Epidemiology of Paediatric *Mycoplasma Pneumoniae* Pneumonia in North China: 2006 to 2016. Epidemiol. Infect. 147, e192. 10.1017/S0950268819000839 31364532 PMC6518602

[B10] Gullsby K. Olsen B. Bondeson K. (2019). Molecular Typing of Mycoplasma Pneumoniae Strains in Sweden From 1996 to 2017 and the Emergence of a New P1 Cytadhesin Gene, Variant 2e. J. Clin. Microbiol. 57, e00049–e00019. 10.1128/JCM.00049-19 30918047 PMC6535615

[B11] Ho P. L. Law P. Y. Chan B. W. Wong C. W. To K. K. Chiu S. S.. (2015). Emergence of Macrolide-Resistant *Mycoplasma Pneumoniae* in Hong Kong Is Linked to Increasing Macrolide Resistance in Multilocus Variable-Number Tandem-Repeat Analysis Type 4-5-7-2. J. Clin. Microbiol. 53, 3560–3564. 10.1128/JCM.01983-15 26338857 PMC4609705

[B12] Hsia Y. Lee B. R. Versporten A. Yang Y. Bielicki J. Jackson C.. (2019). Use of the WHO Access, Watch, and Reserve Classification to Define Patterns of Hospital Antibiotic Use (AwaRe): An Analysis of Paediatric Survey Data From 56 Countries. Lancet Global Health 7, e861–e871. 10.1016/S2214-109X(19)30071-3 31200888

[B13] Hung H. M. Chuang C. H. Chen Y. Y. Liao W. C. Li S. W. Chang I. Y.. (2020). Clonal Spread of Macrolide-Resistant *Mycoplasma Pneumoniae* Sequence Type-3 and type-17 With Recombination on Non-P1 Adhesin Among Children in Taiwan. Clin. Microbiol. Infect. S1198–743X (20), 30588–30597. 10.1016/j.cmi.2020.09.035 33010445

[B14] Kawai Y. Miyashita N. Kubo M. Akaike H. Kato A. Nishizawa Y.. (2013). Nationwide Surveillance of Macrolide-Resistant *Mycoplasma Pneumoniae* Infection in Pediatric Patients. Antimicrob. Agents Chemother. 57, 4046–4049. 10.1128/AAC.00663-13 23716043 PMC3719750

[B15] Kenri T. Okazaki N. Yamazaki T. Narita M. Izumikawa K. Matsuoka M.. (2008). Genotyping Analysis of Mycoplasma Pneumoniae Clinical Strains in Japan Between 1995 and 2005: Type Shift Phenomenon of M. Pneumoniae Clinical Strains. J. Med. Microbiol. 57, 469–475. 10.1099/jmm.0.47634-0 18349367

[B16] Kogoj R. Mrvic T. Praprotnik M. Kese D. (2015). Prevalence, Genotyping and Macrolide Resistance of *Mycoplasma Pneumoniae* Among Isolates of Patients With Respiratory Tract Infections, Central Slovenia, 2006 to 2014. Euro Surveill. 20 (37). 10.2807/1560-7917.ES.2015.20.37.30018 26536357

[B17] Kogoj R. Praprotnik M. Mrvic T. Korva M. Kese D. (2018). Genetic Diversity and Macrolide Resistance of *Mycoplasma Pneumoniae* Isolates From Two Consecutive Epidemics in Slovenia. Eur. J. Clin. Microbiol. Infect. Dis. (37), 99–107. 10.1007/s10096-017-3106-5 28948376

[B18] Lee J. K. Lee J. H. Lee H. Ahn Y. M. Eun B. W. Cho E. Y.. (2018). Clonal Expansion of Macrolide-Resistant Sequence Type 3 *Mycoplasma Pneumoniae*, South Korea. Emerg. Infect. Dis. 24, 1465–1471. 10.3201/eid2408.180081 30014844 PMC6056092

[B19] Morozumi M. Iwata S. Hasegawa K. Chiba N. Takayanagi R. Matsubara K.. (2008). Increased Macrolide Resistance of *Mycoplasma Pneumoniae* in Pediatric Patients With Community-Acquired Pneumonia. Antimicrob. Agents Chemother. 52, 348–350. 10.1128/AAC.00779-07 17954691 PMC2223908

[B20] Morozumi M. Tajima T. Sakuma M. Shouji M. Meguro H. Saito K.. (2020). Sequence Type Changes Associated With Decreasing Macrolide-Resistant Mycoplasma Pneumoniae, Japan. Emerg. Infect. Dis. 26, 2210–2213. 10.3201/eid2609.191575 32818419 PMC7454074

[B21] Okada T. Morozumi M. Tajima T. Hasegawa M. Sakata H. Ohnari S.. (2012). Rapid Effectiveness of Minocycline or Doxycycline Against Macrolide-Resistant Mycoplasma Pneumoniae Infection in a 2011 Outbreak Among Japanese Children. Clin. Infect. Dis. 55, 1642–1649. 10.1093/cid/cis784 22972867

[B22] Pereyre S. Touati A. Petitjean-Lecherbonnier J. Charron A. Vabret A. Bebear C. (2013). The Increased Incidence of Mycoplasma Pneumoniae in France in 2011 was Polyclonal, Mainly Involving M. Pneumoniae Type 1 Strains. Clin. Microbiol. Infect. 19, E212–E217. 10.1111/1469-0691.12107 23279613

[B23] Pneumonia (community-acquired): antimicrobial prescribing (2019) NICE Guideline. Available at: www.nice.org.uk/guidance/ng138.

[B24] Qian Q. Wei J. (2016). Epidemiological Features of Children Hospitalized With Mycoplasma Pneumoniae Infection From the Year of 2006 to 2014. Chongqing Med. 29, 4113–4116. 10.7666/d.D01005406

[B25] Qu J. Yu X. Liu Y. Yin Y. Gu L. Cao B.. (2013). Specific Multilocus Variable-Number Tandem-Repeat Analysis Genotypes of *Mycoplasma Pneumoniae* are Associated With Diseases Severity and Macrolide Susceptibility. PloS One 8, e82174. 10.1371/journal.pone.0082174 24367502 PMC3867324

[B26] Respiratory Branch of Chinese Pediatric Society of Editorial Board of Chinese Journal of Applied Clinical Pediatrics (2015). Expert Consensus on Diagnosis and Treatment of *Mycoplasma Pneumoniae* Pneumonia in Children. Chin. J. Appl. Clin. Pediatr. 30, 1304–1308. 10.3760/cma.j.issn.2095-428X.2015.17.006

[B27] Rivaya B. Jordana-Lluch E. Fernandez-Rivas G. Molinos S. Campos R. Mendez-Hernandez M.. (2020). Macrolide Resistance and Molecular Typing of *Mycoplasma Pneumoniae* Infections During a 4 Year Period in Spain. J. Antimicrob. Chemother. 75, 2752–2759. 10.1093/jac/dkaa256 32653897 PMC7678890

[B28] Subspecialty Group of Respiratory Diseases. The Society of Pediatrics (2013). Chinese Medical Association: Guidelines of Management of Community Acquired Pneumonia in Children (the Revised Edition of 2013). Chin. J. Pediatr. 51, 745–752. 10.3760/cma.j.issn.0578-1310.2013.10.006 24406226

[B29] Sun H. Xue G. Yan C. Li S. Zhao H. Feng Y. (2017). Changes in Molecular Characteristics of *Mycoplasma Pneumoniae* in Clinical Specimens From Children in Beijing Between 2003 and 2015. PloS One 12, e0170253. 10.1371/journal.pone.0170253 28107399 PMC5249184

[B30] Suzuki Y. Seto J. Shimotai Y. Itagaki T. Katsushima Y. Katsushima F.. (2017). Multiple-Locus Variable-Number Tandem-Repeat Analysis of *Mycoplasma Pneumoniae* Isolates Between 2004 and 2014 in Yamagata, Japan: Change in Molecular Characteristics During an 11-Year Period. Jpn. J. Infect. Dis. 70, 642–646. 10.7883/yoken.JJID.2017.276 29093323

[B31] The Committee of Japanese Society of Mycoplasmology (2014). Guiding Principles * for Treating for Treating Mycoplasma Pneumoniae Pneumonia (Tokyo: Japanese Society of Mycoplasmology).

[B32] Uldum S. A. Bangsborg J. M. Gahrn-Hansen B. Ljung R. Molvadgaard M. Fons Petersen R. (2010). Epidemic of Mycoplasma Pneumoniae Infection in Denmark, 2010 and 2011. Euro Surveill. 17, 20073. 10.2807/ese.17.05.20073-en 22321137

[B33] Voronina E. N. Gordukova M. A. Turina I. E. Mishukova O. V. Dymova M. A. Galeeva E. V.. (2020). Molecular Characterization of Mycoplasma Pneumoniae Infections in Moscow From 2015 to 2018. Eur. J. Clin. Microbiol. Infect. Dis. 39, 257–263. 10.1007/s10096-019-03717-6 31655931

[B34] Xiao L. Ratliff A. E. Crabb D. M. Mixon E. Qin X. Selvarangan R.. (2020). Molecular Characterization of *Mycoplasma Pneumoniae* Isolates in the United States From 2012 to 2018. J. Clin. Microbiol. 58, e00710–e00720. 10.1128/JCM.00710-20 32817226 PMC7512161

[B35] Xue G. Li M. Wang N. Zhao J. Wang B. Ren Z.. (2018). Comparison of the Molecular Characteristics of *Mycoplasma Pneumoniae* From Children Across Different Regions of China. PloS One 13, e0198557. 10.1371/journal.pone.0198557 30138360 PMC6107135

[B36] Xue G. Wang Q. Yan C. Jeoffreys N. Wang L. Li S.. (2014). Molecular Characterizations of PCR-Positive *Mycoplasma Pneumoniae* Specimens Collected From Australia and China. J. Clin. Microbiol. 52, 1478–1482. 10.1128/JCM.03366-13 24574282 PMC3993644

[B37] Yang T. I. Chang T. H. Lu C. Y. Chen J. M. Lee P. I. Huang L. M.. (2019). *Mycoplasma Pneumoniae* in Pediatric Patients: Do Macrolide-Resistance and/or Delayed Treatment Matter? J. Microbiol. Immunol. Infect. 52, 329–335. 10.1016/j.jmii.2018.09.009 30341022

[B38] Yan C. Sun H. Xue G. Zhao H. Wang L. Feng Y.. (2014). A Single-Tube Multiple-Locus Variable-Number Tandem-Repeat Analysis of *Mycoplasma Pneumoniae* Clinical Specimens by Use of Multiplex PCR-capillary Electrophoresis. J. Clin. Microbiol. 52, 4168–4171. 10.1128/JCM.02178-14 25232156 PMC4313281

[B39] Yan C. Xue G. Zhao H. Feng Y. Li S. Cui J.. (2019). Molecular and Clinical Characteristics of Severe *Mycoplasma Pneumoniae* Pneumonia in Children. Pediatr. Pulmonol. 54, 1012–1021. 10.1002/ppul.24327 31119869

[B40] Zhao F. Li J. Liu J. Guan X. Gong J. Liu L.. (2019). Antimicrobial Susceptibility and Molecular Characteristics of *Mycoplasma Pneumoniae* Isolates Across Different Regions of China. Antimicrob. Resist. Infect. Control 8, 143. 10.1186/s13756-019-0576-5 31463046 PMC6708159

[B41] Zhao F. Liu G. Cao B. Wu J. Gu Y. He L.. (2013). Multiple-Locus Variable-Number Tandem-Repeat Analysis of 201 *Mycoplasma Pneumoniae* Isolates From Beijing, China, From 2008 to 2011. J. Clin. Microbiol. 51, 636–639. 10.1128/JCM.02567-12 23224090 PMC3553874

[B42] Zhao F. Liu G. Wu J. Cao B. Tao X. He L.. (2013). Surveillance of Macrolide-Resistant *Mycoplasma Pneumoniae* in Beijing, China, From 2008 to 2012. Antimicrob. Agents Chemother. 57, 1521–1523. 10.1128/AAC.02060-12 23263003 PMC3591905

[B43] Zhao F. Lv M. Tao X. Huang H. Zhang B. Zhang Z.. (2012). Antibiotic Sensitivity of 40 *Mycoplasma Pneumoniae* Isolates and Molecular Analysis of Macrolide-Resistant Isolates From Beijing, China. Antimicrob. Agents Chemother. 56, 1108–1109. 10.1128/AAC.05627-11 22106216 PMC3264270

[B44] Zhou Y. Zhang Y. Sheng Y. Zhang L. Shen Z. Chen Z. (2014). More Complications Occur in Macrolide-Resistant Than in Macrolide-Sensitive *Mycoplasma Pneumoniae* Pneumonia. Antimicrob. Agents Chemother. 58, 1034–1038. 10.1128/AAC.01806-13 24277047 PMC3910883

